# MoO_3_ Nanobelts Embedded Polypyrrole/SIS Copolymer Blends for Improved Electro-Mechanical Dual Applications

**DOI:** 10.3390/polym12020353

**Published:** 2020-02-06

**Authors:** Arslan Umer, Faroha Liaqat, Azhar Mahmood

**Affiliations:** 1 School of Natural Sciences, National University of Sciences and Technology, H-12, Islamabad 44000, Pakistan; arslanumer69@gmail.com; 2 Department of Chemistry, Quaid-i-Azam University, Islamabad 45320, Pakistan

**Keywords:** polypyrrole, poly(styrene-isoprene-styrene), nanobelts, conductivity, tensile testing

## Abstract

This research endeavor aimed to develop thin film blends of polypyrrole (PPy) and poly (styrene-isoprene-styrene) (SIS) with MoO_3_ as a nanofiller for improved mechanical and electrical properties to widen its scope in the field of mechatronics. This study reports blends of polypyrrole (PPy) and poly (styrene-isoprene-styrene) (SIS) tri-block copolymer showing improved mechanical and electrical attributes while employing MoO_3_ nanobelts as nanofillers that additionally improves the abovementioned properties in the ensuing nanocomposites. The synthesis of PPy/SIS blends and MoO_3_/PPy/SIS nanocomposites was well corroborated with XRD, SEM, FTIR, and EDS analysis. Successful blending of PPy was yielded up to 15 *w*/*w*% PPy in SIS, as beyond this self-agglomeration of PPy was observed. The results showed a remarkable increase in the conductivity of insulating SIS copolymer from 1.5 × 10^−6.1^ to 0.343 Scm^−1^ and tensile strength up to 8.5 MPa with the 15 *w*/*w*% PPy/SIS blend. A further enhancement of the properties was recorded by embedding MoO_3_ nanobelts with varying concentrations of the nanofillers into 15 *w*/*w*% PPy/SIS blends. The mechanical strength of the polymeric nanocomposites was enhanced up to 11.4 MPa with an increase in conductivity up to 1.51 Scm^−1^ for 3 *w*/*w*% MoO_3_/PPy-SIS blends. The resultant product exhibited good potential for electro-mechanical dual applications.

## 1. Introduction

The last decade has seen an increased interest in the field of conducting polymers (CPs), like polyacetylene (PA), polythiophene (PTh), polypyrrole (PPy), polyparaphenylene (PPPh), polyaniline (PANI), and polyorthotoluidine (POT), due to their use in a variety of applications, such as sensors, light weight batteries, solar cells, transistors, anti-static coatings, compact capacitors, and electromagnetic shielding for various devices [1,2,3,4,5,6,7,8,9,10,11]. Among different CPs, polypyrrole received special interest because of its biocompatibility, cost efficiency, and excellent environmental stability along with controllable electrical conductivity as compared to other CPs [12]. Despite its remarkable properties, PPy exhibits poor thermal properties, insolubility due to cross linking of PPy chains, and lack of mechanical and film-forming properties caused by a rigid structure that limits its usage and large-scale production [13,14]. In order to overcome these limitations of PPy, different modifications are being explored by researchers, including synthesis of different nanohybrid conducting polymeric structures (NHCPs), interpenetrating polymer networks (IPNs), or making blends with other polymers, copolymers, or nanofillers that have superior solubility, stability, process ability, mechanical properties, film-forming capabilities, and electrical conductivity [8,15,16,17,18,19].

Hsing-Lin Wang and Jack E. Fernandez reported that blends of PPy with polyvinyl methyl ketone (PVMK) increased the threshold conductivity up to 10% via an increase of the concentration of PPy content and thermal stability up to 325 °C [20]. Shengyi Zhang et al. successfully synthesized selenium/PPy nanocomposite to enhance the water solubility and conductivity of PPy up to four times at room temperature [21]. Electrochemically synthesized composites of PVC and PPy have also been reported, which generated flexible and free-standing films with improved mechanical strength along with the stability of PPy under ambient conditions [22]. Moreover, the synthesis of water-soluble composites of PPy/polyvinyl alcohol/graphene oxide (GO) via the solution blending method resulted in an increase in the tensile strength of PPy/PVA/GO blends and amplified electrical properties that made them suitable for electronic applications [23]. Keeping the aforementioned studies in view, it can be anticipated that limitations of polypyrrole could be overcome by exploring the development of its various composites and blends.

In this research communication, the authors report the synthesis of polypyrrole/poly (styrene-isoprene-styrene) blends and their nanocomposites, with MoO_3_ acting as a nanofiller. Resultant materials were subjected to various characterization techniques, including FTIR, SEM/EDS, and XRD while their electrical and mechanical properties were also investigated. To the best of our knowledge, these MoO_3_ nanobelt-embedded composites with blends of PPy/poly (SIS) have not been investigated earlier.

## 2. Materials and Methods

All the materials viz. aqueous solution of 99% pure pyrrole (Daejung, Seoul, Korea), anhydrous ferric chloride (Sigma Aldrich, Taufkirchen, Germany), ammonium molybdate (Merck, Darmstadt, Germany), methanol (Daejung, Seoul, Korea), acetone (Daejung, Seoul, Korea), chloroform (Daejung, Seoul, Korea), nitric acid (70%, Daejung, Seoul, Korea), and tetrahydrofuran (Daejung, Seoul, Korea) were obtained from commercial sources. Poly(styrene-isoprene-styrene) was obtained (Sigma Aldrich, Taufkirchen, Germany), containing 22% tyrene by weight. All chemicals were of reagent grade and used without further purification.

### 2.1. Synthesis of Polypyrrole by Chemical Polymerization

As reported elsewhere, the chemical oxidative polymerization method was used to synthesize polypyrrole from its monomer using FeCl_3_ as an oxidant [24]. Anhydrous FeCl_3_ (2 g) was added in 50 mL of chloroform at 25 °C and stirred for 20 min. Subsequently, pyrrole (10 mL) was added dropwise to this solution under continuous stirring at 35 °C. Upon the addition of the first drop of pyrrole, the light orange color of the solution turned black. Polymerization was allowed to proceed for 3 h under vigorous stirring. Finally, filtration, washing, and drying furnished black powdered polypyrrole.

### 2.2. Synthesis of PPy Blend with SIS Copolymer

Blends of polypyrrole/poly (styrene-isoprene-styrene) were synthesized via the dispersion blending method. Both polymers were dispersed in a common THF solvent (5–20 *w*/*w*%) with continuous stirring to facilitate uniform dispersion of PPy and SIS in the solvent. After 24 h of vigorous stirring at 40 °C, these blends were left overnight to allow maximum solvent evaporation. However, some THF was trapped in the films, which was removed by heating at 45 °C in a vacuum oven for 32 h. This resulted in the synthesis of PPy/SIS blend films with the composition of 5, 10, 15 and 20 *w*/*w*%. It was observed that the PPy/SIS 20 *w*/*w*% blend did not exhibit a uniform dispersion of PPy in SIS and showed agglomeration.

### 2.3. Synthesis of MoO_3_ Nanobelts

MoO_3_ nanobelts were synthesized by the hydrothermal method [25]. In total, 1 g of ammonium molybdate ((NH_4_)_6_Mo_7_O_24_) was added into about 30 mL of deionized water under continuous stirring. After one hour, 5 mL of nitric acid (HNO_3_) were added dropwise into the mixture and stirred for another hour. Subsequently, the resulting suspension was transferred into a 50-mL Teflon-lined autoclave and heated at 180 °C for 24 h. Finally, the product was filtered, washed, and dried in a vacuum oven to obtain pure MoO_3_ nanobelts.

### 2.4. Synthesis of Polymeric Nanocomposites of MoO_3_/PPy-SIS Copolymer

Nanocomposites of MoO_3_ nanobelts with PPy-SIS blends were also synthesized via the dispersion blending technique while using THF as a solvent. Respective blends were sonicated for 4 h at 35 °C followed by 24 h of stirring. The complete dispersion solutions were mixed and annealed overnight in a petri dish to evaporate the solvent. The resultant film was completely dried at 45 °C in a vacuum oven. In this way, MoO_3_ nanobelts were embedded as reinforcement into the polymer blends, which augmented the phase properties of the PPy-blend-SIS matrix. PPy-SIS blend films with 1, 2, 3, and 4 *w*/*w*% MoO_3_ nanobelts were synthesized.

### 2.5. Instrumentations and Methods

The resultant synthesized materials (PPy, MoO_3_, PPy/SIS, MoO_3_/PPy-SIS) were characterized by Fourier transform infrared spectroscopy (FTIR) using an Alpha (BRUKER) spectrophotometer in the range of 4000 to 500 cm^−1^ and X-ray diffraction analysis (XRD) using an X-ray diffractometer (D8 advance BRUKER) equipped with a CuKα radiation source, with a wavelength of 0.154 nm and a graphite monochromator in a 2θ range of 10 to 80°. The morphology, size, and composition of synthesized materials were investigated via scanning electron microscope coupled with energy dispersive X-ray spectroscope (SEM/EDS) (VEGA3 TESCAN) at an accelerating voltage of 20 kV. The mechanical properties of the prepared blends were studied according to ASTM D882 by using a SHIMADZU tensile testing machine at the rate of 5 mm/min. Four probe conductivities were measured by the JANDEL RM3000 test unit in order to investigate the conducting properties of the synthesized nanocomposites while keeping the current constant.

## 3. Results and Discussions

### 3.1. FTIR Analysis of PPy, PPy/SIS Blends, and MoO_3_/PPy-SIS Nanocomposites

**Figure 1:**
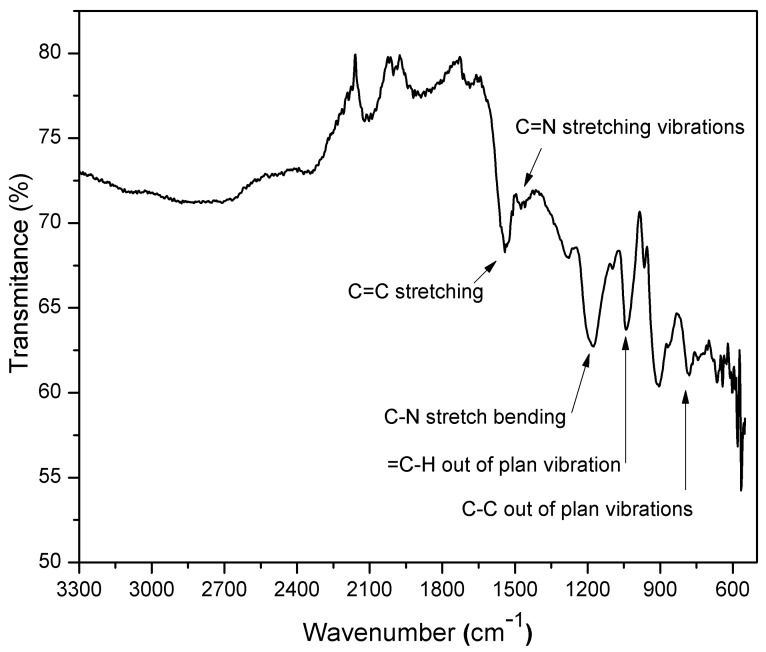
FTIR spectrum of pure polypyrrole.

Figure 1 depicts the FTIR spectrum of the as-synthesized pure PPy. The characteristic bands of protonated PPy were observed at 1541 and 1458 cm^−1^ due to C=C and C=N stretching vibrations of the ring, respectively [26]. Absorption bands at 1175 (C-N stretch bending), 1041 (=C-H out of plan vibration), 965, 904 (C-H out of plan deformation vibration), 780 (C-C out of plan vibrations), and 665 cm^−1^ (C-H out of plan vibrations) also corroborated the PPy structure [27].

**Figure 2:**
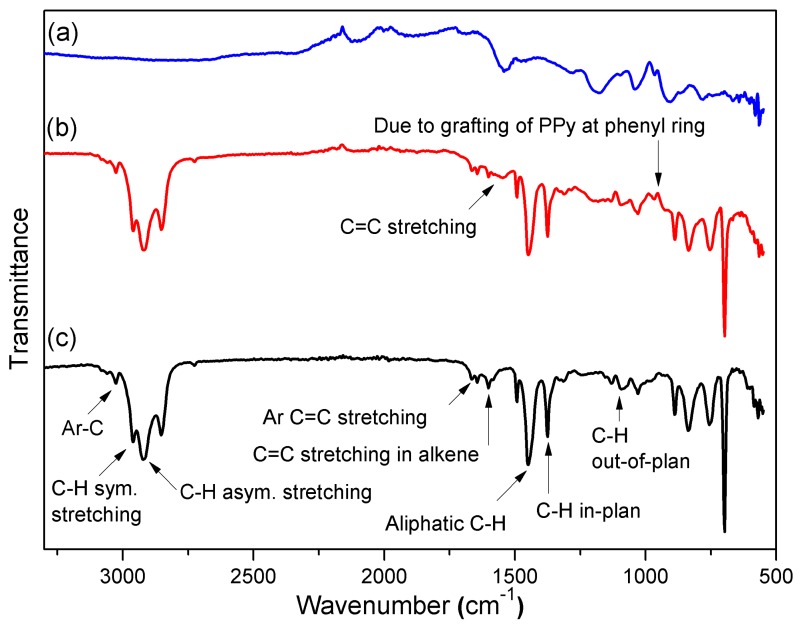
FTIR spectra of: (**a**) Pure polypyrrole (PPy); (**b**) 15 *w*/*w*% PPy/SIS Blends; (**c**) Pure poly (styrene-isoprene-styrene) (SIS).

The FTIR spectrum of pure SIS copolymer is shown in Figure 2c. The aromatic ring of polystyrene in SIS exhibited vibrations at 3030 cm^−1^. The bands at 2977 and 2916 cm^−1^ were due to C-H symmetrical and C-H asymmetrical stretching in the aromatic ring, respectively, while C=C stretching in the aromatic ring, C-H bonding in the aliphatic chain, and C=C stretching in alkene were illustrated at 1648, 1441, and 1595 cm^−1^, respectively. C-H in-plane and out-of-plane stretching vibrations were observed at 1373 and 1014 cm^−1^.

Figure 2b depicts the FTIR spectrum of 15 *w*/*w*% PPy/SIS blends. Although the FTIR spectra of 5 and 10 *w*/*w*% PPy/SIS blends were recorded, they did not exhibit significant differences due to the low concentration of PPy. Figure 2b shows certain changes in the absorption region of pure SIS, i.e., a broad band at 1541 cm^−1^ and a new band at 964 cm^−1^, due to the grafting of PPy at the phenyl ring in the SIS copolymer. This confirmed the successful incorporation of polypyrrole into the SIS copolymer.

**Figure 3:**
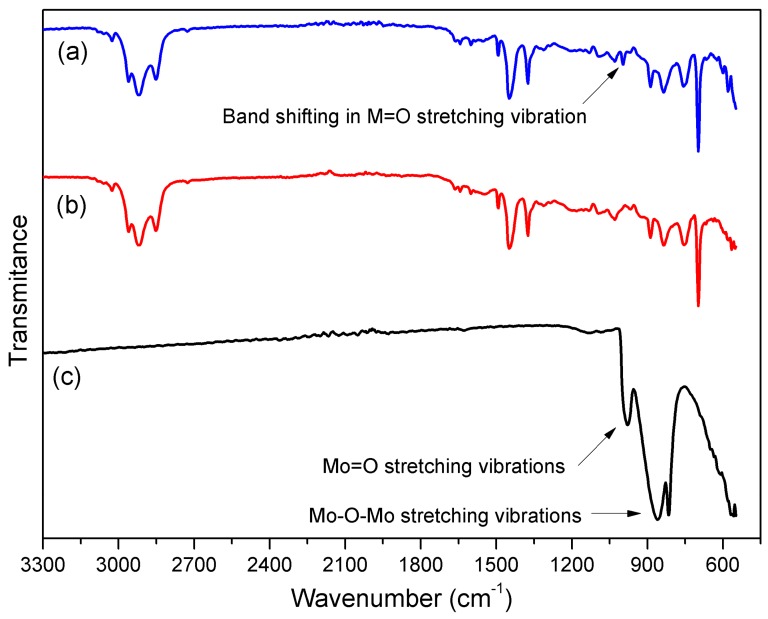
FTIR spectra of MoO_3_/PPy/SIS blends: (**a**) MoO_3_/PPy/SIS blends; (**b**) 15 *w*/*w*% PPy/SIS blend; (**c**) Pure MoO_3_ nanobelts.

A typical band for MoO_3_ (978 cm^−1^) (Figure 3c) was shifted to 997 cm^−1^ in the ensuing MoO_3_/PPy-SIS blend (Figure 3a) while certain bands in the region of 540 to 580 cm^−1^, as seen in Figure 3a, revealed the presence of MoO_3_ nanobelts in the PPy/SIS blends.

### 3.2. Morphology and Elemental Composition of MoO_3_ Nanobelts

**Figure 4:**
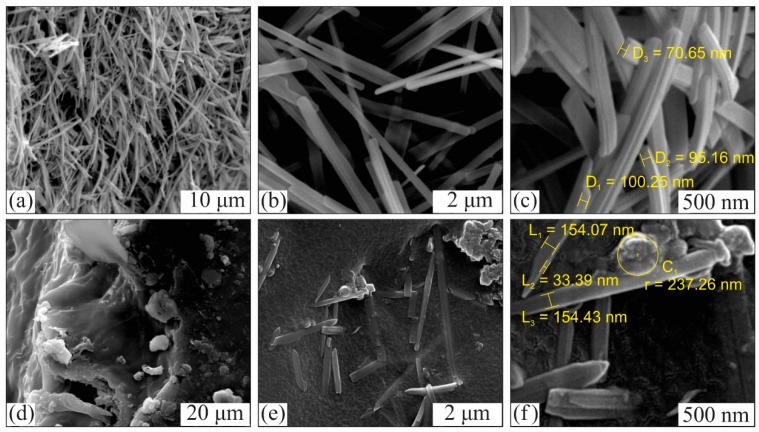
SEM images: (**a**–**c**) MoO_3_ nanobelts; (**d**) cross section of PPy/SIS blend; (**e**,**f**) MoO_3_ nanobelts incorporated in the PPy and SIS matrix forming nanocomposites.

To investigate the morphology and structure of the synthesized MoO_3_ nanobelts, SEM images were recorded at different magnifications while the elemental composition was determined via EDS. Micrographs of the MoO_3_ and EDS results revealed a uniform nanobelt morphology without any impurity or aggregates. The length of the nanobelts was measured up to 5 to 10 µm. Figure 4b shows that the nanobelts have a rectangular cross section instead of a round one, with a uniform width of 150 to 200 nm. Moreover, Figure 4c shows that at certain points, a few nanobelts were piled over one another, forming layered structures. Accurate measurement of their thickness was not possible as these belts were lying flat on the support due to the small thickness. However, it was estimated to be 60 to 100 nm on the basis of the recorded SEM images.

Figure 4d depicts an irregularly broken edge in the PPy/SIS blends that confirmed the incorporation of PPy molecules in the SIS copolymer matrix while Figure 4e,f depict a homogeneous distribution of MoO_3_ nanobelts in the polymeric matrix and their intercalation with incorporated PPy particles to serve as a nanofiller in PPY/SIS nanocomposites.

**Figure 5:**
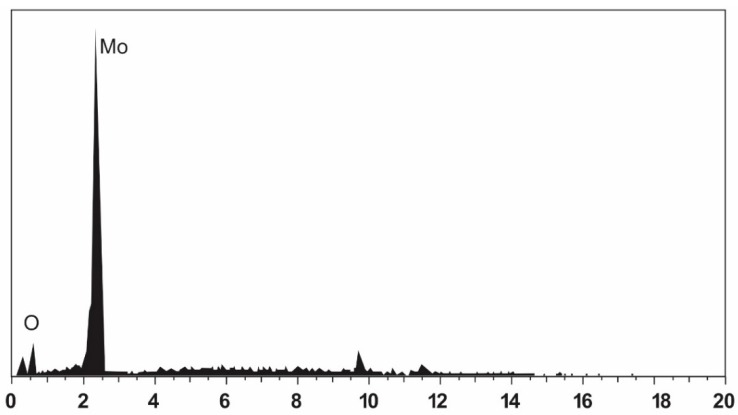
Energy-dispersive X-ray spectrum of MoO_3_ nanobelts.

The elemental composition of the MoO_3_ nanobelts was determined via EDS analysis (Table 1 and Figure 5). The composition of MoO_3_ nanobelts was found to be 74.82 atomic weight % for oxygen and 25.18 atomic weight % for molybdenum, which further verified the formation of pure MoO_3_ nanobelts. Although a carbon peak also appeared in the EDS spectrum, it could be attributed to the grid.

### 3.3. XRD Analysis

**Figure 6:**
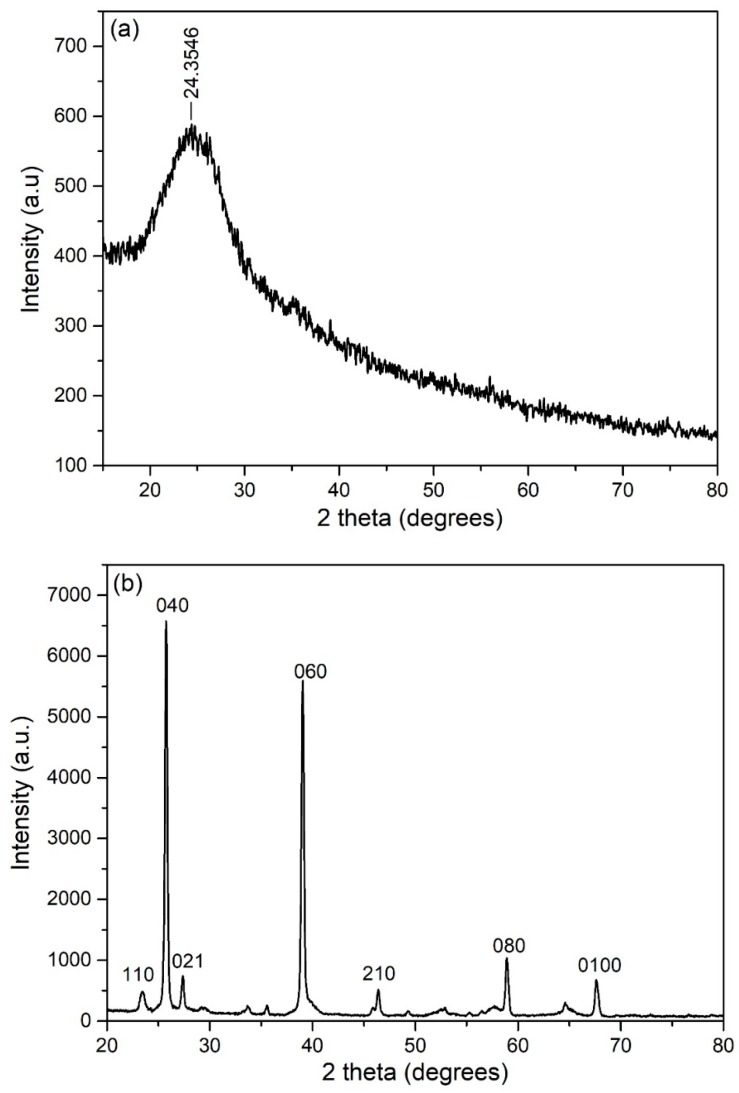
XRD patterns of: (**a**) Pure PPy; (**b**) MoO_3_ nanobelts.

The XRD pattern in Figure 6a confirms the amorphous nature of pure PPy powder. The characteristic peak of polypyrrole was observed at (2θ) 24.35°, which indicated the short-range arrangement of PPy chains [27]. The value of d spacing for PPy was measured to be 3.6599 A°. The crystallographic information of MoO_3_ nanobelts as well as the phase purity was also investigated through XRD analysis. The diffraction peaks of MoO_3_ nanobelts in Figure 6b were perfectly indexed to orthorhombic α-MoO_3_ (JCPDS card no. 05-0508). The high purity of the sample is indicated from an absence of any noticeable impurity peak. The high intensity of 020, 040, and 060 diffraction peaks conformed the highly anisotropic growth of nanobelts.

**Figure 7:**
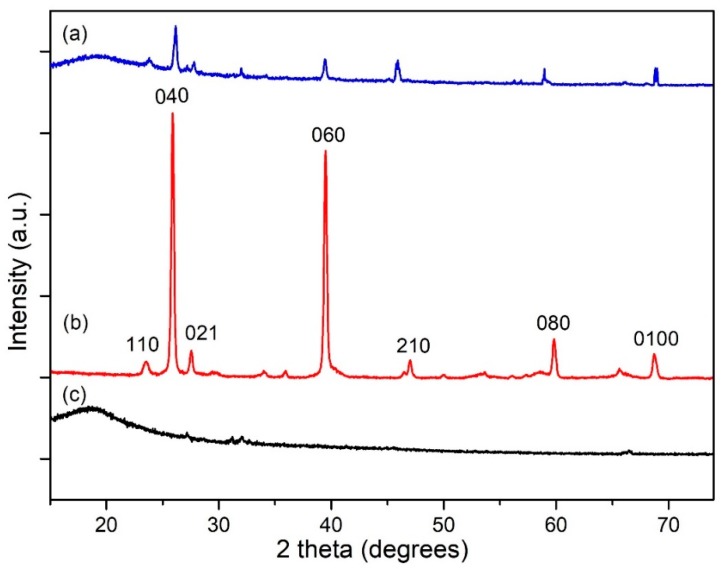
XRD patterns showing the incorporation of MoO_3_ nanobelts in 15 *w*/*w*% PPy/SIS blend: (**a**) MoO_3_/PPy/SIS nanocomposite; (**b**) MoO_3_ nanobelts; (**c**) PPy/SIS blend (15 *w*/*w*%).

Figure 7 depicts the XRD changes that occurred by the incorporation of MoO_3_ nanobelts into 15 *w*/*w*% PPy/SIS blends. Distinctive peaks that appeared at approximately 32° in Figure 7c correspond to the PPy/SIS blend (15 *w*/*w*%) and the 040 and 060 planes in Figure 7b correspond to MoO_3_ nanobelts, as shown in Figure 7a, which shows the XRD of the resultant nanocomposites. Moreover, in the XRD spectra of both the PPy/SIS blends and MoO_3_/PPy/SIS nanocomposites, a slight increase in intensity near 20° confirmed the presence of PPy.

### 3.4. Conductivity Measurements

Electrical conductivity properties of MoO_3_/PPy/SIS nanocomposites with varying compositions were studied by the four-probe technique. The electrical resistivity of different *w*/*w*% of MoO_3_/PPy/SIS nanocomposites was measured by passing a constant current of 1 µA through the films at room temperature. The resistivity values were converted into electrical conductivity by taking the reciprocal values (conductivity = 1/resistivity).

**Figure 8:**
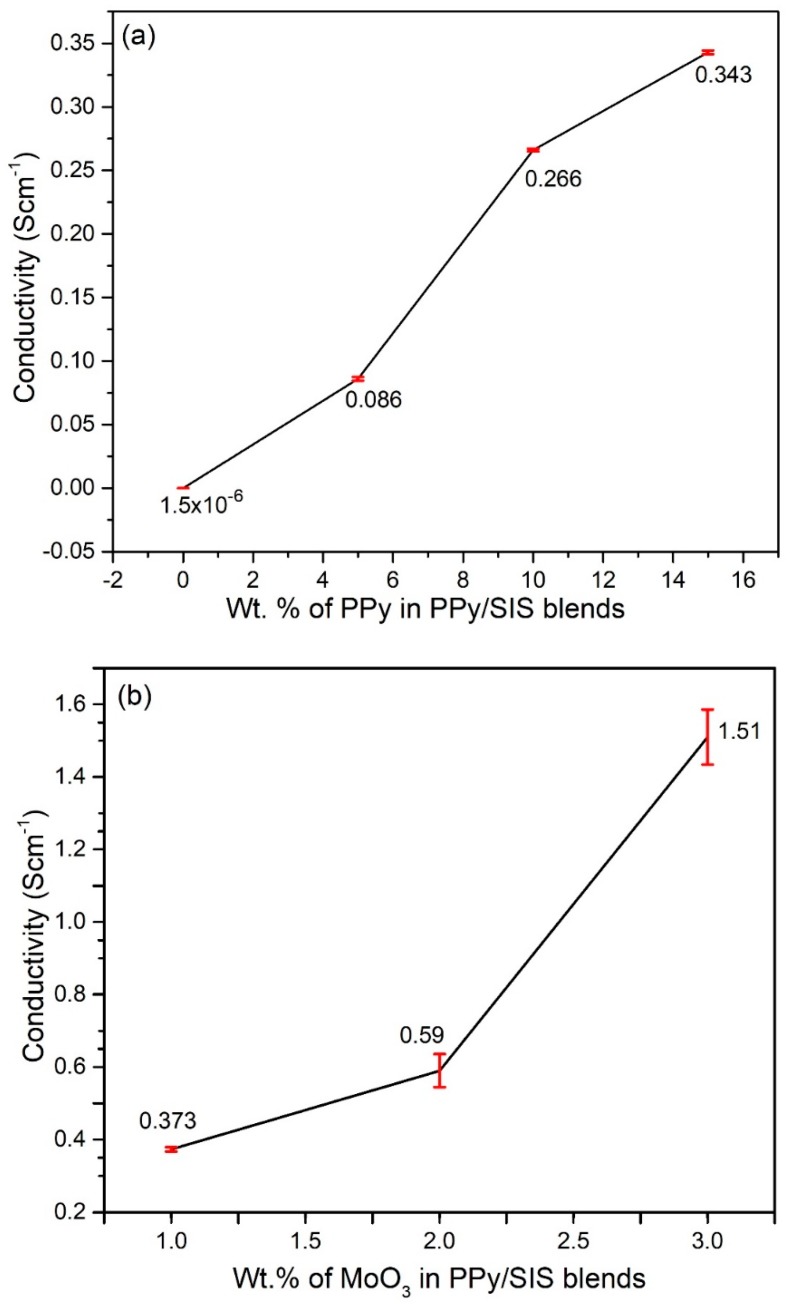
Electrical conductivity trend with variation in: (**a**) Amount (*w*/*w* %) of PPy in the PPy/SIS blends; (**b**) Amount of MoO_3_ nanobelts (1, 2, and 3 *w*/*w* %) in a selected polymer blend (15 *w*/*w*% PPy/SIS).

Figure 8a shows the electrical conductivity of various PPy/SIS blends without the incorporation of MoO_3_ nanobelts. A pure SIS film exhibits poor conductivity in the order of 1.5 × 10^−6^ Scm^−1^, but the electrical conductivity was enhanced significantly to 0.086 Scm^−1^ by making blends with 5 *w*/*w*% of PPy. This was further increased up to the optimum value of 0.343 Scm^−1^ by the addition of 15 *w*/*w*% of PPy. The 15 *w*/*w*% PPy in the SIS matrix exhibited the best results. Above this *w*/*w*% concentration of PPy, the conductivity of blends decreased due to the self-agglomeration of PPy.

MoO_3_ nanobelts were added as nanofillers to study the effect of nanobelts on the electrical properties of 15 *w*/*w*% PPy/SIS blends. The conductivity of the MoO_3_/15 *w*/*w*% PPy/SIS nanocomposite film was measured as 0.373, 0.59, and 1.51 Scm^−1^ on the addition of 1, 2, and 3 *w*/*w*% of MoO_3_ nanobelts, respectively, which is even greater than the reported conductivity of pure PPy itself (Figure 8b) [28]. A large increase in conductivity was observed with 3 *w*/*w*% nanobelts. It is proposed that MoO_3_ nanobelts may act as conducting junctions between PPy chains that are incorporated in the SIS matrix [29]. As a result, multiple polarons can be generated and a wide range of localized energy states are created. This causes particular distortions in the polymer backbone, causing an increase in the conductivity of PPy/SIS nanocomposites [29]. However, upon further enrichment of MoO_3_ (i.e., 4 *w*/*w*%) into the PPy/SIS blend, the conductivity decreases due to the agglomeration of MoO_3_ nanobelts in the PPy/SIS matrix.

### 3.5. Mechanical Properties

**Figure 9:**
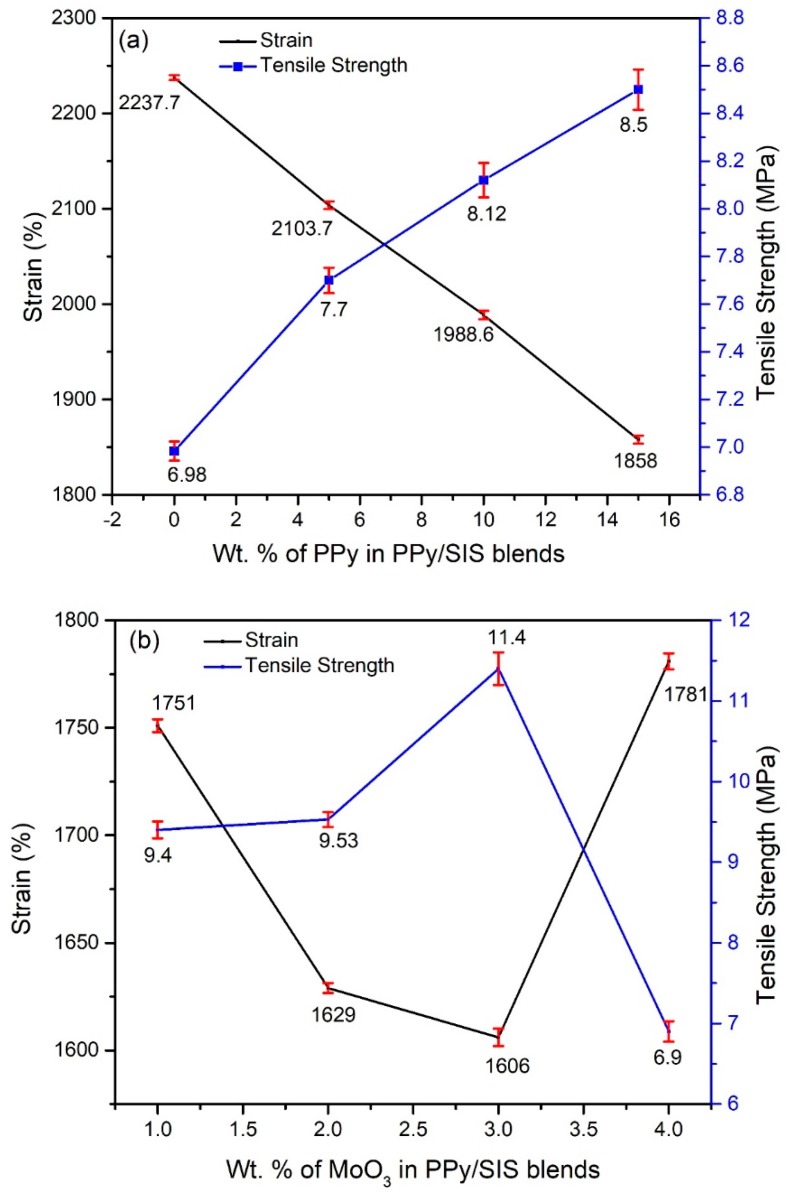
Tensile strength and % strain of various blends: (**a**) 0, 5, 10, and 15 *w*/*w*% of PPy/SIS blends; (**b**) 1, 2, 3, and 4 *w*/*w*% of MoO_3_/PPy/SIS nanocomposites.

The mechanical properties of the synthesized films and nanocomposites were studied as per the ASTM D882 standard. The tensile strength of the blends was determined by the maximum force required to break the film at the breaking point area under the stress–strain curve [30]. The change in the mechanical properties of SIS on the addition of different *w*/*w*% PPy is well explained by the tensile strength and strain curves (Figure 9). Pure SIS shows the highest strain (2237.7%) with the least tensile strength value (6.98 MPa) in Figure 9a and Table 2. This indicates its elastic nature and little resistance towards deformation. As 5 *w*/*w*% PPy was incorporated into pure SIS, the tensile strength increased up to 7.70 MPa with a decrease in the strain to 2103.7%. This indicates an increased resistance toward deformation due to the fact that the brittle nature of PPy prevents the elongation of SIS [31]. Moreover, a dramatic decrease in the area under the stress–strain curve of this blend shows that less energy is required to break the film. The tensile strength of the 15 *w*/*w*% PPy/SIS blend increased up to 8.50 MPa while elongation decreased to 1858.0%. The Young’s modulus value also increased with increasing *w*/*w*% of PPy in the SIS copolymer, from 0.800 to 1.217 MPa as thee composition was changed from 0 to 15 *w*/*w*% PPy/SIS blends. This increase in tensile strength and Young’s modulus indicates the improvement in the strength and stiffness up to the 15 *w*/*w*% PPy/SIS blend. Agglomeration was observed when the polypyrrole content was further increased up to 20 *w*/*w*%.

In order to study the effect of nanobelts on the mechanical properties, MoO_3_ nanobelts were added as nanofillers within the range of 1 to 4 *w*/*w*% into the 15 *w*/*w*% PPy/SIS blend. The effect of nanofiller on Young’s modulus, % strain, and tensile strength was also measured and reported (Figure 9b and Table 3). It can be seen that embedded nanobelts further improve the mechanical properties of 3 *w*/*w*% MoO_3_/PPy/SIS nanocomposite film by limiting elongation up to 1606% and elevating the Young’s modulus as well as tensile strength up to 2.170 and 11.40 MPa respectively. However, upon further enrichment of MoO_3_ into the PPy/SIS blend, the Young’s modulus and tensile strength decreased due to the self-agglomeration of MoO_3_ nanobelts in the PPy/SIS matrix. Thus, optimum mechanical properties were exhibited by the 3 *w*/*w*% MoO_3_/PPy/SIS nanocomposite film.

## 4. Conclusions

Polypyrrole and poly(styrene-isoprene-styrene) nanocomposite films with improved electrical and mechanical features were successfully developed while employing MoO_3_ nanobelts as nanofillers. The synthesis of blends and nanocomposites was well corroborated with the XRD, FTIR, SEM, and EDS analysis data. Various proportions of PPy (0, 5, 10, 15, and 20 *w*/*w*%) in the SIS matrix were explored to optimize the composition of the blend to enhance the processability of infusible PPy. Successful blending of PPy was yielded up to 15 *w*/*w*% PPy, as beyond this, self-agglomeration of PPy was observed. These blends were subjected to electrical, morphological, and mechanical studies by the four-probe conductivity technique, SEM, and a tensile testing machine, respectively. The results showed a remarkable increase in the conductivity of insulating SIS copolymer from 1.5 × 10^−6^ to 0.343 Scm^−1^ and tensile strength up to 8.5 MPa with the 15 *w*/*w*% PPy/SIS blend.

Further enhancement in the electrical and mechanical properties was observed by embedding MoO_3_ nanobelts with varying concentrations (1–3 *w*/*w*% of MoO_3_) into 15 *w*/*w*% PPy/SIS blends as nanofillers. The mechanical strength was enhanced up to 11.4 MPa with the increased conductivity up to 1.51 Scm^−1^ for 3 *w*/*w*% MoO_3_/PPy-SIS nanocomposites. Morphological studies of these MoO_3_/PPy/SIS nanocomposites via SEM revealed a homogeneous dispersion of MoO_3_ nanobelts in the PPy/SIS blends. Therefore, it is proposed that MoO_3_ nanobelts may act as conducting junctions between PPy chains that increase the electrical conductivity and modulus of the resultant nanocomposite films.

## Figures and Tables

**Table 1:** Elemental composition of MoO_3_ nanobelts by EDS.

Element	Weight %	Atomic %
Oxygen	33.13	74.82
Molybdenum	66.87	25.18

**Table 2:** Mechanical properties of different *w*/*w*% PPy/SIS blends.

*w*/*w* % of PPy in SIS	% Strain	Tensile Strength (MPa)	Young Modulus (MPa)
0	2237.7	6.98	0.800
5	2103.7	7.70	1.027
10	1988.6	8.12	1.081
15	1858.0	8.50	1.217

**Table 3:** Mechanical properties of different *w*/*w*% MoO_3_/PPy/SIS nanocomposites.

*w*/*w* % of PPy in SIS	% Strain	Tensile Strength (MPa)	Young Modulus (MPa)
1	1751	9.40	1.580
2	1629	9.53	1.830
3	1606	11.40	2.170
4	1781	6.90	1.442
